# Magnetic resonance imaging-guided radiotherapy for intermediate- and high-risk prostate cancer: Trade-off between planning target volume margin and online plan adaption

**DOI:** 10.1016/j.phro.2022.06.013

**Published:** 2022-07-03

**Authors:** Shyama U. Tetar, Anna M.E. Bruynzeel, Lisa Verweij, Omar Bohoudi, Berend J. Slotman, Tezontl Rosario, Miguel A. Palacios, Frank J. Lagerwaard

**Affiliations:** Department of Radiation Oncology, Amsterdam University Medical Centers, Location VUmc, de Boelelaan 1117, 1081 HV Amsterdam, The Netherlands

**Keywords:** Prostate cancer, Seminal vesicles, MR-guided radiotherapy, Interfraction motion, Plan adaptation, Dosimetry

## Abstract

Magnetic resonance-guided radiotherapy with daily plan adaptation for intermediate- and high-risk prostate cancer is time and labor intensive. Fifty adapted plans with 3 mm planning target volume (PTV)-margin were compared with non-adapted plans using 3 or 5 mm margins. Adequate (V95% ≥ 95%) prostate coverage was achieved in 49 fractions with 5 mm PTV without plan adaptation, however, coverage of the seminal vesicles (SV) was insufficient in 15 of 50 fractions. There was no insufficient coverage for prostate and SV using plan adaptation with 3 mm. Hence, daily adaptation is recommended to obtain adequate SV-coverage when using 3 mm PTV.

## 1 Introduction

Modern radiation therapy for localized prostate cancer (PC) is characterized by the increasing trend towards (ultra-)hypofractionation and use of image-guided radiotherapy (IGRT) [1]. Most commonly, IGRT setup involves the combination of cone-beam computed tomography (CBCT) and implanted fiducial markers. This approach allows for smaller margins which have been shown to reduce acute rectal toxicity compared to setup on the bony anatomy [2], [3]. However, the application of increasingly smaller margins in combination with hypofractionation underscores the necessity to ensure adequate target coverage. Whereas high-frequency IGRT during delivery based on implanted fiducial markers may be well able to safeguard coverage of the prostate [4], [5], the seminal vesicles (SV) exhibit greater inter- and intrafractional positional variation [6], [7], [8], [9].

Current delineation guidelines for clinical target volume (CTV) definition advise inclusion of at least 1.4 cm and 2.2 cm of the proximal SV for intermediate- and high-risk PC, respectively [10]. New imaging techniques such as multiparametric magnetic resonance imaging (mpMRI) and prostate-specific membrane antigen positron emission tomography (PSMA-PET) are increasingly being implemented in the clinical staging of PC. However, the sensitivity and interobserver variation for determining SV infiltration remain not well defined [11], [12], [13]. To date, this newer diagnostic information has not yet altered the aforementioned guidelines for PC radiotherapy target definition.

MR-guided radiotherapy (MRgRT) with or without daily plan adaptation is a relatively novel addition to the arsenal of techniques for (ultra-)hypofractionated treatment of localized PC [14], [15], [16]. MR-based soft-tissue imaging prior to each fraction provides detailed visualization of the target volume and organs at risk (OAR), and can therefore be used to determine dosimetric coverage of the prostate and SV separately. Daily online adaptation requires recontouring, rapid replanning and quality assurance of the new treatment plan and allows optimal coverage of both the prostate and SV. The aim of this study was to criticize the necessity of (time- and labor intensive) daily online plan adaptation for coverage of the prostate and SV, by comparing this with non-adapted treatment plans using 3 and 5 mm safety margins, respectively.

## 2 Materials and methods

Clinical and imaging data of patients treated with MRgRT at our center are stored in a prospective institutional review board approved database (IRB 2018_602). This analysis was performed in ten patients with intermediate or high risk PC, treated with 36.25 Gy in 5 fractions with daily adaptive MRgRT on the MRIdian (ViewRay Inc., Mountain View, USA). Details of our workflow are described in the Supplementary material. All 10 patients underwent a MRI (0.35 T; TrueFISP) at simulation and prior to delivery of each fraction. Treatment plans were optimized ensuring ≥95% of the planning target volume (PTV) covered with 95% of the prescribed dose. Planning objectives and normal tissue constraints are presented in Supplementary table 1.

Separate dosimetric coverage of the prostate and SV was evaluated by partitioning CTV’s in each available MRI-scans per patient, thus generating prostate-CTV’s (CTV_PR-BASELINE_, CTV_PR-Fx1-5_) and seminal vesicles-CTV (CTV_SV-BASELINE_, CTV_SV-FX1-5_). Contouring of the CTV was performed following the ESTRO ACROP consensus guidelines, i.e. prostate and proximal 1.4 cm and 2.2 cm of the SV for intermediate- and high-risk, respectively [10]. In case of cT3b disease, the whole SV was included in the CTV. An isotropic CTV, including CTV_PR_ and CTV_SV_, to PTV margin of 3 mm was used, as described previously [17]. Contouring was performed by the same radiation oncologist. For each patient, multiple plans were generated; a) baseline plan (PLAN_BASELINE-3mm_), b) non-adapted baseline plans recalculated on the anatomy for each fraction for both 3 mm and 5 mm CTV to PTV margins, for both CTV_PR_ and CTV_SV_ (PLAN_RECALC-3mm_ and PLAN_RECALC-5mm_, respectively) and c) adapted plans for each fraction (PLAN_REOPT-3mm_).

Individual fraction CTV coverage was defined as “sufficient” (≥95% of CTV receiving 95% of prescribed dose) or “insufficient” (<95% of CTV receiving 95% of prescribed dose). Dose coverage of the CTV_PR_ and CTV_SV_ and volumes of the rectum and bladder receiving ≥ 36.25 Gy were measured in all generated plans for each fraction.

### 2.1 Statistical analysis

Statistical analysis was performed using IBM SPSS Statistics for Windows, version 26 (IBM Corp., Armonk, N.Y., USA). Wilcoxon signed-rank test was used for 1) evaluating differences between the CTV_PR_ and CTV_SV_ coverage for all different plans for each fraction and, 2) evaluating differences between volumes of the rectum and bladder receiving ≥ V36.25 Gy. A *p*-value < 0.05 was considered statistically significant.

## 3 Results

### 3.1 Baseline plans

All ten baseline plans met the institutional criteria for target coverage and OAR constraints, with a median V95% of 99.5% and 99.8% for the CTV_PR-BASELINE_ and CTV_SV-BASELINE_, respectively. The median V36.25 Gy was 0.29 cc and 0.11 cc for the bladder and rectum, respectively.

### 3.2 Prostate coverage for each fraction

**Fig. 1:**
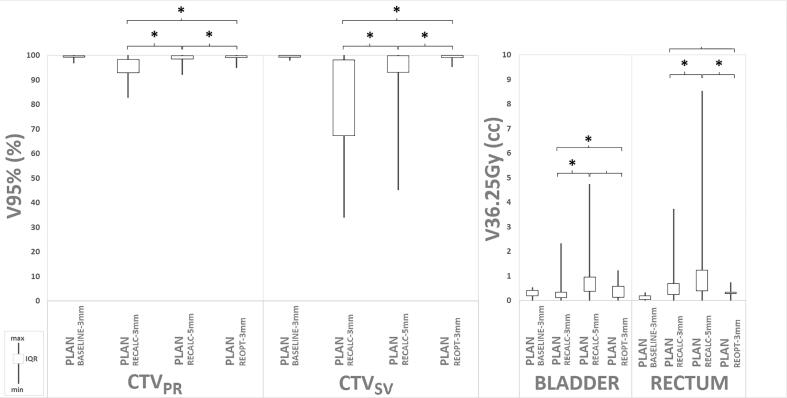
Boxplots for median V95% coverage to prostate and seminal vesicles in compared plans (left). Boxplots for V36.25 Gy to organs at risk in compared plans (right). CTV_PR_ = clinical target volume of prostate. CTV_SV_ = clinical target volume of seminal vesicles. * = statiscally significant with p-value < 0.05.

The median V95% of CTV_PR-RECALC-3mm_, CTV_PR-RECALC-5mm_ and CTV_PR-REOPT-3mm_ were 96.1%, 99.2%, and 99.5%, respectively. In general, the coverage of CTV_PR_ was statistically significant higher in both PLAN_RECALC-5mm_ and PLAN_REOPT-3mm_ with respect to PLAN_RECALC-3mm_ (*p* < 0.001). In addition, PLAN_PR-REOPT-3mm_ exhibited an improved coverage in comparison to PLAN_RECALC-5mm_ (*p* = 0.017). Fig. 1 presents the box-plots and (non-)significances of the V95% for CTV_PR_ in all plans. More importantly, whereas after re-optimization no plans were observed with “insufficient” CTV_PR_ coverage, this was the case in 17 of 50 (34%) of PLAN_RECALC-3mm_ and 1 of 50 (2%) of PLAN_RECALC-5mm_, respectively (Supplementary table 2).

### 3.3 SV coverage for each fraction

The median V95% of CTV_SV-RECALC-3mm_, CTV_SV-RECALC-5mm_ and CTV_SV-REOPT-3mm_ were 83.6%, 98.9% and 99.5%, respectively. Both PLAN_RECALC-5mm_ (p < 0.001) and PLAN_REOPT-3mm_ (p < 0.001) were statistically superior compared to PLAN_RECALC-3mm_. Again, PLAN_REOPT-3mm_ was statistically superior (p < 0.001) when compared with PLAN_RECALC-5mm_. Fig. 1 presents the box-plots and (non-)significances of the V95% for CTV_SV_ in all plans. None of the 50 PLAN_REOPT-3mm_ showed “insufficient” CTV_SV_ coverage. In contrast, “insufficient” CTV_SV_ coverage was seen in 15/50 fractions (30%) and 29/50 fractions (58%) in PLAN_RECALC-5mm_ and PLAN_RECALC-3mm_, respectively.

### 3.4 Organs at risk doses

The median bladder V36.25 Gy of PLAN_RECALC-3mm_ was 0.12 cc, whereas it was 0.29 cc for PLAN_RECALC-5mm_ and 0.39 cc for PLAN_REOPT-3mm_. The corresponding median rectal V36.25 Gy values were 0.19 cc (PLAN_RECALC-3mm_), 0.37 cc (PLAN_RECALC-5mm_) and 0.14 cc (PLAN_REOPT-3mm_). Fig. 1 presents the OAR box-plots and (non-)significances of the V36.25 Gy for all plans.

## 4 Discussion

In our study of ten patients with intermediate- and high-risk prostate cancer, daily plan adaptation (PLAN_REOPT-3mm_) showed the most optimal coverage for both the prostate and the SV compared to non-adapted treatment using either 3 or 5 mm safety margins. For coverage of prostate-CTV, applying 5 mm margins seems to be sufficient (98% of plans), however at the cost of higher doses to the surrounding organs. With respect to the coverage of the SV-CTV, the effect of daily adaptation is even more clear. Non-adapted plans with a 5 mm margin (PLAN_RECALC-5mm_) led to an increase of the fractions with sufficient coverage for the SV with respect to the use of 3 mm margins (PLAN_RECALC-3mm_). However, even this larger PTV-margin, did not result in sufficient coverage of all fractions, making online plan re-optimization necessary. Our results underscore previous findings of Ma et al., who concluded that online adaptive therapy may be indicated to account for prostatic swelling and in particular proximal SV rotations [18]. Their conclusion was based on interfraction volumetric changes in the prostate and proximal SV with 2 mm PTV-margins, which showed a V95% ≥ 95% coverage was achieved in 94% of fractions for the prostate and in only 59% for proximal SV.

SV tumor involvement is associated with poorer biochemical failure-free survival, metastasis-free survival and overall survival [19], [20]. A study on patterns of local failure in 284 men evaluated with post-radiation biopsy after a median time of 61 months, showed most recurrences at initial dominant tumor sites. Of 140 patients with mapped pre- and post-treatment biopsies, 4% demonstrated cancer in a new location previously identified as negative [21]. Recommendations to include the seminal vesicles in the recent ESTRO guidelines for target volume delineation for primary radiotherapy of localized PC [10] are based on pretreatment clinical tumor characteristics i.e. PSA, Gleason score, clinical T-stage and involved positive biopsies [22], [23], on surgical series [24] and the 3D imaging analysis of Qi et al [25].

**Fig. 2:**
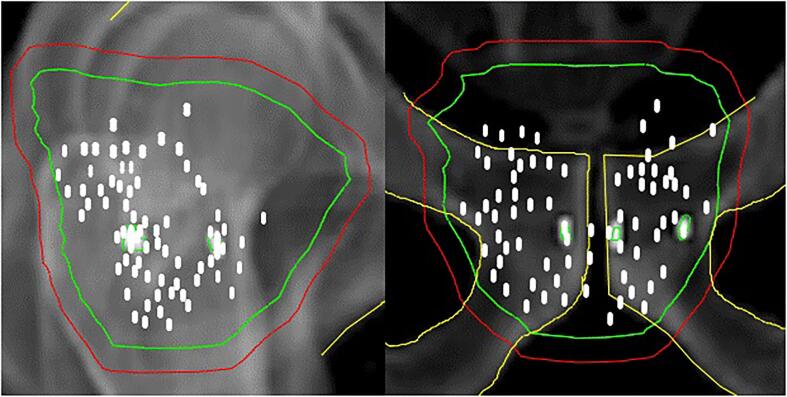
Locations of implanted fiducial markers in 23 patients need for set-up on a regular linac. Green = clinical target volume. Red = planning target volume.

IGRT for PC patients is usually performed using implanted fiducial markers in the prostate. Safety margins to account for set-up errors and target motion could therefore be reduced and consequently, reduce the dose to OARs. The combination of IGRT techniques and smaller PTV-margins allows safe dose escalation to the target. A cohort study of 2142 men with low- and intermediate-risk PC treated with stereotactic body radiotherapy showed low rates of severe toxic events after a median follow- up of 7 years [26]. On the contrary, smaller PTV-margins could also lead to geometrical target miss due to intra-fraction motion and large inter-fraction rotations. In a retrospective study, 50 patients were treated with a small anisotropic PTV-margin of 3–5 mm or with an isotropic PTV-margin of 6 mm using daily IGRT on implanted markers. The authors of the study reported an increased freedom from biochemical failure at 5-year using 6 mm PTV-margin compared to the tighter PTV margins (p = 0.04) [27]. Current ESTRO guidelines on IGRT for localized PC ensures prostate coverage with intraprostatic markers, but not necessarily SV coverage. Recommendation is therefore based on various target margins for prostate and SV separately, with a larger margin on SV, based on prostate matching [3]. A review of 23 prostate cancer patients treated with EBRT with cone-beam setup at our institution demonstrated that none of the fiducials were placed in the vesicles (Fig. 2).

A recent review shows that inter- and intrafraction motion of SV is substantial and largely uncorrelated with prostate motion and is influenced mainly by rectal and bladder filling. Translations, rotations, deformations and volume changes need to be taken into account for calculating PTV-margins. In contrast to inter-fraction motion, intra-fraction motion is more complex and stresses the need for tracking and/or soft tissue gating [28].

There are some limitations of this study to be mentioned in addition to the relatively limited number of 50 fractions that has been studied. Rigid MR-registrations to obtain recalculated plans were performed with a focus on the prostate and less on the SV. Rotations were not performed during registrations on the CTV, because MR-linacs doesn’t allow 6D couch corrections. Furthermore, intra-fractional anatomical changes were not taken into account in this study. This could occur during time-consuming adaptive process as well as during radiotherapy treatment delivery. Mannerberg et al. reported on CTV underdosage of 1.1%, 2.0% and 4.2% when using a PTV margin of 7 mm, 5 mm and 3 mm, respectively, occurring in a 30 min timeframe necessary for adaptive re-planning [29]. In addition, they reported a mean center of mass vector offset for the CTV of 1.92 mm [0.13–9.79 mm] caused by bladder volume increase and rectum volume difference [29]. Another study on SV intrafraction motion on the MR-Linac used 3D Cine-MRI during 10 min beam-on time. SV motion shows a larger variation than prostate motion, and moreover increases over time especially in anterior and cranial directions [30].

One strength of this study is the reduction of interobserver variables, with a single radiation oncologist consequently contouring all fractions. In addition, the use of MRgRT improves visualization of seminal vesicles, compared to previous CT/CBCT based studies and could lead to more precise SV target definition and dosimetric evaluation [31].

With the introduction of MRgRT and daily plan adaptation, the precision and accuracy of radiotherapy delivery for PC is improved, which has led to the application of smaller safety margins. Several studies have emphasized adaptive radiotherapy for adequate SV coverage across all fractions [32], [33]. In our study on interfraction motion, we have demonstrated that non-adapted plans using 3 mm results in insufficient coverage of the prostate as well as the SV. Target coverage is improved by applying a larger 5 mm margin but still one third had insufficient coverage for SV-CTV and moreover at the expense of a higher dose to the rectum. Therefore, we conclude that the application of a tighter PTV-margin of 3 mm can only be safely performed when using daily plan adaptation to ensure coverage for SV, and in a lesser extent for the prostate.

## Declaration of Competing Interest

The authors declare that they have no known competing financial interests or personal relationships that could have appeared to influence the work reported in this paper.
