# Supplementary material for: Magnetic resonance imaging-guided radiotherapy for intermediate- and high-risk prostate cancer: Trade-off between planning target volume margin and online plan adaption
Source: Phys Imaging Radiat Oncol. 2022 Jul 3;23:92–6. doi: 10.1016/j.phro.2022.06.013 (PMC9283928; doi:10.1016/j.phro.2022.06.013)
Supplement: Supplementary Data 1 [file mmc1.docx]

**Supplementary material**

**Workflow for daily adaptive MR-guided radiotherapy on the MRIdian**

During our MRgRT workflow patients underwent a simulation MRI (0.35T; TrueFISP sequence; TR/TE: 3.37ms/1.45 ms; FA 60°; resolution 1.5mm x 1.5mm x 1.5mm) and a simulation CT-scan (slice thickness 2 mm), co-registered to the MRI for dose-calculation purposes. Patients instructions included aiming for a half-full bladder and empty rectum. The prescription dose to the planning target volume (PTV) was 36.25 Gy in 5 fractions delivered within two weeks. Treatment plans were optimized with step-and-shoot IMRT ensuring at least a 95% of the PTV with 95% of the prescribed dose, i.e. 34.43 Gy. Prior to delivery of each fraction, volumetric MRI was repeated, followed by recontouring of the CTV and OAR, plan re-optimization and plan quality assurance. The planning protocol used for baseline planning and daily re-optimization, including objectives and constraints for target volumes and OAR are shown in Supplementary table 1.

***Supplementary table 1:*** *Dose prescription for target coverage and normal tissue constraints
Organs at risk are only re-contoured within 2 cm of the prostate and for adaptive setting only dose in these structured are optimized. Abbreviations: PTV = planning target volume. CTV = clinical target volume*

| Structure | **Dose to volume** | | | |
| --- | --- | --- | --- | --- |
| PTV prostate | ≥ 95 | % at | 34.4 | Gy |
| PTV prostate | ≤ 2 | % at | 39.9 | Gy |
| CTV prostate | ≥ 98 | % at | 34.4 | Gy |
| Rectum in 2 cm | ≤ 0.1 | cc at | 38.1 | Gy |
|  | ≤ 1 | cc at | 36.3 | Gy |
|  | ≤ 5 | cc at | 34.4 | Gy |
|  | ≤ 10 | cc at | 32.6 | Gy |
| Bladder in 2 cm | ≤ 0.1 | cc at | 37.0 | Gy |
|  | ≤ 1 | cc at | 36.3 | Gy |
|  | ≤ 15 | cc at | 32.6 | Gy |
| Bowel in 2 cm | ≤ 0.1 | cc at | 36.0 | Gy |
|  | ≤ 1 | cc at | 33.0 | Gy |

***Supplementary table 2******:*** *Median CTV coverage of prostate and seminal vesicles in all plans, including number of fractions with sufficient (*V95% ≥ 95%) *and insufficient (*V95% < 95%) *coverage. Abbreviations: CTV = clinical target volume. CTV_PR_ = clinical target volume of prostate. CTV_SV_ = clinical target volume of seminal vesicles.*

| *CTV coverage by 95% of the dose* | PLAN_BASELINE-3mm_ | PLAN_RECALC-3mm_ | PLAN_RECALC-5mm_ | PLAN_REOPT-3mm_ |
| --- | --- | --- | --- | --- |
| CTV_PR_ (n=10) |  |  |  |  |
| Median  (range) | 99.5%  (97.0-99.8) | 96.1%  (83.2-99.7%) | 99.2%  (92.2-99.9%) | 99.5%  (95.0-100%) |
| ‘sufficient’ fractions |  | 66% (N=33) | 98% (N=49) | 100% (N=50) |
| ‘insufficient’ fractions |  | 34% (N=17) | 2% (N=1) | 0% (N=0) |
| CTV_SV_ (n=10) |  |  |  |  |
| Median  (range) | 99.8%  (98.0-100%) | 83.6%  (34.1-99.9%) | 98.9%  (45.4-100%) | 99.5%  (95.5-100%) |
| ‘sufficient’ fractions |  | 42% (N=21) | 70% (N=35) | 100% (N=50) |
| ‘insufficient’ fractions |  | 58% (N=29) | 30% (N=15) | 0% (N=0) |
